# Vestibular feedback maintains reaching accuracy during body movement

**DOI:** 10.1113/JP273125

**Published:** 2016-11-13

**Authors:** Craig P. Smith, Raymond F. Reynolds

**Affiliations:** ^1^ School of Sport, Exercise and Rehabilitation SciencesUniversity of BirminghamUK

**Keywords:** galvanic vestibular stimulation, upper‐limb control, vestibular system

## Abstract

**Key points:**

- Reaching movements can be perturbed by vestibular input, but the function of this response is unclear.
- Here, we applied galvanic vestibular stimulation concurrently with real body movement while subjects maintained arm position either fixed in space or fixed with respect to their body.
- During the fixed‐in‐space conditions, galvanic vestibular stimulation caused large changes in arm trajectory consistent with a compensatory response to maintain upper‐limb accuracy in the face of body movement.
- Galvanic vestibular stimulation responses were absent during the body‐fixed task, demonstrating task dependency in vestibular control of the upper limb.
- The results suggest that the function of vestibular‐evoked arm movements is to maintain the accuracy of the upper limb during unpredictable body movement, but only when reaching in an earth‐fixed reference frame.

**Abstract:**

When using our arms to interact with the world, unintended body motion can introduce movement error. A mechanism that could detect and compensate for such motion would be beneficial. Observations of arm movements evoked by vestibular stimulation provide some support for this mechanism. However, the physiological function underlying these artificially evoked movements is unclear from previous research. For such a mechanism to be functional, it should operate only when the arm is being controlled in an earth‐fixed rather than a body‐fixed reference frame. In the latter case, compensation would be unnecessary and even deleterious. To test this hypothesis, subjects were gently rotated in a chair while being asked to maintain their outstretched arm pointing towards either earth‐fixed or body‐fixed memorized targets. Galvanic vestibular stimulation was applied concurrently during rotation to isolate the influence of vestibular input, uncontaminated by inertial factors. During the earth‐fixed task, galvanic vestibular stimulation produced large polarity‐dependent corrections in arm position. These corrections mimicked those evoked when chair velocity was altered without any galvanic vestibular stimulation, indicating a compensatory arm response to a sensation of altered body motion. In stark contrast, corrections were completely absent during the body‐fixed task, despite the same chair movement profile and arm posture. These effects persisted when we controlled for differences in limb kinematics between the two tasks. Our results demonstrate that vestibular control of the upper limb maintains reaching accuracy during unpredictable body motion. The observation that such responses occurred only when reaching within an earth‐fixed reference frame confirms the functional nature of vestibular‐evoked arm movement.

## Key points

- Reaching movements can be perturbed by vestibular input, but the function of this response is unclear.
- Here, we applied galvanic vestibular stimulation concurrently with real body movement while subjects maintained arm position either fixed in space or fixed with respect to their body.
- During the fixed‐in‐space conditions, galvanic vestibular stimulation caused large changes in arm trajectory consistent with a compensatory response to maintain upper‐limb accuracy in the face of body movement.
- Galvanic vestibular stimulation responses were absent during the body‐fixed task, demonstrating task dependency in vestibular control of the upper limb.
- The results suggest that the function of vestibular‐evoked arm movements is to maintain the accuracy of the upper limb during unpredictable body movement, but only when reaching in an earth‐fixed reference frame.

**Abbreviation**
ACW: anticlockwise
BF: body fixed
CW: clockwise
EF: earth fixed
GVS: galvanic vestibular stimulation

## Introduction

When reaching for an object, it is necessary to compensate for any body movement that may take the limb off target. The ability to correct arm movements in this way is a common requirement, e.g. reaching for a hand rail while standing on an accelerating train. It requires continuous processing of hand and target position, comparing the predicted outcome of the movement with the intended target location and correcting for any error (Desmurget *et al*. 1998; Desmurget & Grafton, 2000). Even without visual feedback, it is possible to maintain accuracy when reaching to a memorized earth‐fixed target during body motion (Bresciani *et al*. 2002 *c*, 2005). In this case, vestibular signals may provide the sensory input used to compensate for body movement (for review, see Blouin *et al*. 2015).

Evidence linking vestibular input to control of the upper limb has come from the use of galvanic vestibular stimulation (GVS; Bresciani *et al*. 2002 *a*,*b*; Mars *et al*. 2003; Pu *et al*. 2012; Moreau‐Debord *et al*. 2014). Galvanic vestibular stimulation artificially stimulates the vestibular system, producing a false sensation of movement, primarily consisting of head roll (Day *et al*. 1997; Day & Cole, 2002; Marsden *et al*. 2002; Wardman *et al*. 2003; Day & Fitzpatrick, 2005; Reynolds & Osler, 2012). When GVS is applied simultaneously with real motion, subjects perceive that they have moved further or less, depending on the electrode polarity (Fitzpatrick *et al*. 2002; Day & Fitzpatrick, 2005). This effect is modulated by head orientation, being greatest when the naso‐occipital axis is aligned with the axis of rotation, i.e. with the head tilted up or down during yaw motion (rotation about a vertical axis; Day & Fitzpatrick, 2005). In addition to altering perception, GVS can evoke movement. When standing, it induces sway towards the anode electrode, compensating for a sense of body displacement towards the cathode (Lund & Broberg, 1983; Day *et al*. 1997; Marsden *et al*. 2002; Fitzpatrick & Day, 2004). When reaching for a memorized earth‐fixed target, GVS deviates hand trajectory towards the anodal ear (Bresciani *et al*. 2002 *a*,*b*; Mars *et al*. 2003; Moreau‐Debord *et al*. 2014). Similar to the postural response, the arm response has been interpreted as compensation for sensed whole‐body displacement (Bresciani *et al*. 2002 *a*,*b*; Moreau‐Debord *et al*. 2014). Moreau‐Debord *et al*. (2014) demonstrated that, exactly as for the postural system, the effect of GVS upon arm movement is systematically modulated by head orientation, with the largest effect being observed with the head pitched downward. In this case, the stimulus evokes a sense of yaw motion, resulting in a compensatory leftward (or rightward) arm movement.

Nonetheless, the precise nature of the link between vestibular input and upper‐limb motor output is unclear from previous research. Arm movements evoked by GVS have been reported to be of similar magnitude when seated *vs*. standing (Bresciani *et al*. 2002 *a*,*b*). This contrasts with findings from the postural literature, where responses to GVS are normally suppressed when sitting (Britton *et al*. 1993; Fitzpatrick *et al*. 1994; Day & Cole, 2002; Day & Reynolds, 2005; Blouin *et al*. 2007). For instance, lower‐limb responses are absent when seated (Britton *et al*. 1993; Fitzpatrick *et al*. 1994). Even when sitting quietly with the trunk unsupported (i.e. sat on a stool), GVS‐evoked trunk movement is minimal compared with when standing (Day & Cole, 2002; Day & Reynolds, 2005; Blouin *et al*. 2007). The reasons for this suppression are likely to be twofold. Firstly, vestibular‐evoked balance responses are task dependent; when the balance task is minimized by sitting or lying down, vestibular information is not relevant in the same way, and GVS‐evoked leg muscle activity disappears (Britton *et al*. 1993; Fitzpatrick *et al*. 1994). Secondly, the control of balance involves integration of multiple sensory inputs. Reliance upon vestibular input therefore depends upon the availability of information from other modalities. Removing vision or proprioception increases GVS response magnitude (Britton *et al*. 1993; Fitzpatrick *et al*. 1994; Welgampola & Colebatch, 2001; Bent *et al*. 2002; Day & Cole, 2002; Day *et al*. 2002). Conversely, addition of tactile information suppresses the response. When sitting or lying down, all of these factors come into play; the balance task is abolished (or at least minimized), and tactile information from the chair offers an additional source of sensory information. This would explain the reduced GVS responses (Britton *et al*. 1993; Fitzpatrick *et al*. 1994; Day & Cole, 2002; Day & Reynolds, 2005; Blouin *et al*. 2007). Hence, if the arm‐movement response to GVS is produced to compensate for a sensed whole‐body motion, one would expect it to exhibit similar suppression to postural responses, when sitting *vs*. standing. But this is not observed (Bresciani *et al*. 2002 *a*,*b*). Given this discrepancy between arm movements *vs*. sway responses, the underlying mechanism linking vestibular input to the upper limb remains unclear.

Here, we determine whether vestibular input can help to maintain upper‐limb accuracy by compensating for whole‐body motion. If this compensatory mechanism is truly functional, it should operate only when the arm is being moved in an earth‐fixed (EF) reference frame. Such compensation would be unnecessary, and would even impair movement accuracy, when operating in a body‐fixed (BF) reference frame. We therefore compare the effect of GVS upon the arm when performing tasks in both reference frames.

## Methods

Participants were required to point with their arm while seated in a rotating chair. Two experiments were performed to investigate the effect of vestibular feedback upon upper‐limb movement. In both experiments, GVS was applied simultaneously with real motion to isolate the effect of pure vestibular input upon arm movement, unaffected by inertial forces. The purpose of experiment 1 was to investigate the task dependency of the GVS response. This involved a comparison of pointing in a body‐fixed *vs*. an earth‐fixed reference frame. Given that arm kinematics differed between these two tasks, experiment 2 was designed to control for this difference. Additionally, experiment 2 allowed the effect of GVS to be compared against real changes in rotation amplitude.

### Ethical approval

Ethical approval was obtained from the University of Birmingham Ethics Committee and was in compliance with the *Declaration of Helsinki*. Informed written consent was obtained from all participants.

### Subjects

Eight subjects completed experiment 1 (27.8 ± 7.4 years old; five males and three females) and experiment 2 (29.8 ± 5.8 years old; six males and two females). Subjects were healthy, with no known history of vestibular or neurological disorders.

### Apparatus

**Figure 1:**
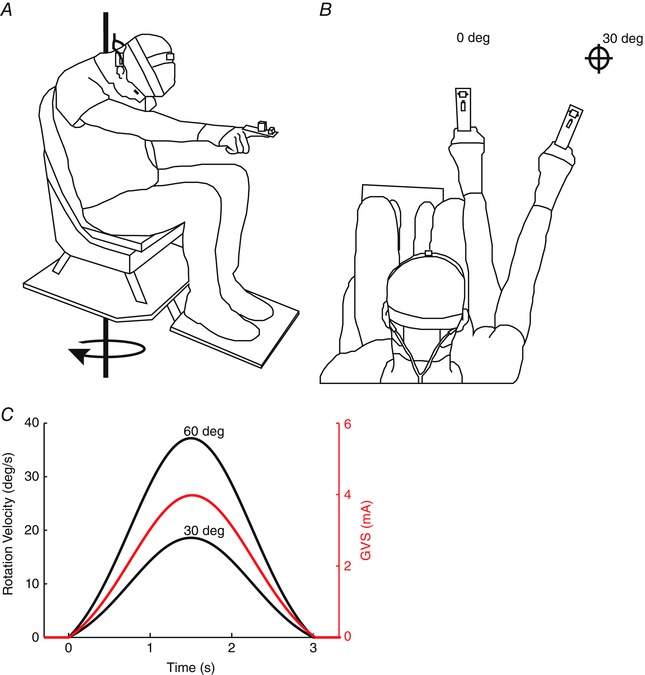
Experimental set‐up *A*, the subject is seated on a rotating chair, with motion‐tracking sensors attached to the head and finger splint. A laser pointer on the splint provides visual feedback at the beginning of each trial when pointing to the target. A wrist support ensures that the hand and forearm move *en bloc*. Galvanic vestibular stimulation (GVS) is applied via electrodes placed over the mastoid processes. *B*, starting position of the arm in the body‐fixed task (0 deg) and earth‐fixed task (30 deg) during experiment 1. *C*, chair rotation profiles are shown for both rotation amplitudes alongside GVS current profile.

The experimental set‐up is illustrated in Fig. 1 *A*. Subjects sat on a motor‐driven rotating chair, which could be controlled with a precision of <1 deg (SD of displacement). They were secured by a four‐point harness. Chair position and velocity were controlled and sampled at 1 kHz using Real Time Windows Target in Simulink (Mathworks Inc., Natick, MA, USA). A motion‐tracking system (Fastrak; Polhemus Inc., Colchester, VT, USA) recorded arm and head movement at 33.33 Hz. The arm sensor was placed over the fingertip, securely fixed to the end of a splint on the right index finger to minimize finger movement. A wrist support minimized wrist movement. During the period when vision was available at the beginning of each trial, subjects used a laser pointer attached to the same finger to guide their arm to the starting position. The head sensor was attached to the top of a welding helmet frame worn by the subject. Fastrak Euler angles were used to derive arm yaw and head pitch (for further details, see Reynolds, 2011).

Galvanic vestibular stimulation currents were applied using gel‐coated carbon rubber electrodes (46 mm × 37 mm) placed over the mastoid processes in a binaural bipolar configuration. Stimuli were delivered from an isolated constant‐current stimulator (model 2200; A‐M Systems, Sequim, WA, USA). Peak current amplitude was 4 mA for all trials, regardless of chair rotation velocity; hence, the effects of chair rotation amplitude could be analysed in isolation.

### Protocol

#### Experiment 1

The purpose of experiment 1 was to determine whether the effect of GVS depends upon the reference frame in which the arm is controlled. Earth‐fixed *vs*. body‐fixed reference frame tasks were studied separately using a blocked design.

During EF trials, the subjects were instructed to point directly at a target attached to the wall of the room, situated 30 deg to their right (Fig. 1 *B*). At the beginning of each trial, they used the laser pointer to align their arm to this target. They then closed their eyes and tilted their head down as far as was comfortable, in order to maximize the effect of vestibular stimulation upon rotation perception. The mean angle of Reid's plane (a line joining the external auditory meatus and inferior orbital margin on each side) was 45.23 ± 6.02 deg below horizontal. After 2 s of baseline recording, the chair was then rotated with a Gaussian velocity profile (Fig. 1 *C*). Their task was simply to maintain the hand pointing towards the memorized target throughout the movement. Two magnitudes of chair rotation were used; 30 (peak velocity = 18.6 deg s^−1^) and 60 deg (peak velocity = 37.2 deg s^−1^), in both clockwise (CW) and anticlockwise (ACW) directions. The magnitude and direction were randomized. The duration of chair rotation was always 3.07 s. A further 2 s of data were recorded after rotation cessation. In two‐thirds of randomly selected trials, GVS was applied simultaneously during chair rotation. The velocity signal used to drive the chair was used for the GVS stimulus, scaled to deliver a peak current of 4 mA (see Fig. 1 *C*). The GVS polarity was randomly switched to provide an equal number of left and right cathodal trials.

The BF trials were the same except for task instruction. Subjects were instructed to point straight ahead at the beginning of each trial and to maintain the arm fixed with respect to their body throughout the movement.

Visual feedback of final arm position was not allowed for any conditions.

This design resulted in 24 conditions, as follows: two tasks (EF and BF) × two directions (CW and ACW) × three GVS (no GVS, cathode left and cathode right) × two rotation magnitudes (30 and 60 deg). Three repeats of each condition were performed, resulting in 72 trials.

#### Experiment 2

Experiment 1 is affected by a simple confound; the EF task requires active arm‐on‐body movement, which is absent during the BF task. Hence, any difference in results might not be attributable to the different reference frames *per se*, but the different movement kinematics. Experiment 2 was designed to address this confound by matching arm‐on‐body movement between tasks.

For the BF task, subjects started by pointing towards a target 60 deg to their right. When they felt chair motion, they were instructed to move their arm to their body mid‐line (i.e. in front of their chest). They were asked approximately to match the duration of arm movement with that of chair movement. In this way, the arm movement was approximately synchronized with chair rotation, whilst being performed in body co‐ordinates. For the EF task, subjects were instructed to point at the same starting target. Rather than simply maintaining the arm position here, as in experiment 1, they were asked to produce a small movement within an earth‐fixed frame. Specifically, when chair rotation started they moved the arm from the 60 deg start position to a target 45 deg to their right. This ensured that some movement was performed within the EF reference frame. Chair rotation was restricted to 60 deg (peak velocity = 37.6 deg s^−1^) and the CW direction (experiment 1 showed no CW–ACW differences). Galvanic vestibular stimulation was applied in two‐thirds of randomly selected trials, in the same way as for experiment 1. The head was tilted downward in a similar manner and eyes were closed throughout all trials. The mean angle of Reid's plane was 67.24 ± 13.34 deg below horizontal. The design of the BF and EF tasks resulted in very well‐matched movement kinematics, in terms of peak arm‐on‐body velocity (see grey bars in Fig. 6 *D*).

This resulted in a total of six conditions, as follows: two tasks (EF and BF) × three GVS (no GVS, cathode left and cathode right). Ten trials per condition were performed, resulting in 60 trials in total.

An obvious problem when attempting to match arm‐on‐body movement between EF and BF tasks, as described above, is to ascertain whether a person is following instructions, i.e. how does the experimenter know the person is genuinely attempting to point in the correct reference frame, if the movements look outwardly similar? To address this issue, we included a block of trials at the beginning of experiment 2, without GVS, where the amplitude of chair rotation was randomly altered (between 50, 60 and 70 deg, equating to peak velocities of:31.3, 37.6 and 43.9 deg s^−1^, always CW). Our rationale was that this alteration should affect arm movement very differently between EF and BF tasks, but only if subjects are correctly following instructions. Specifically, rotation amplitude should alter arm‐on‐body movement only if moving the arm in an EF reference frame. Data from subjects who could not perform the tasks correctly (i.e. exhibit significant effect of rotation amplitude in the BF task) were excluded from further analysis. An additional benefit was to enable direct comparison of GVS‐evoked arm movements with those evoked by real changes in rotation amplitude.

This resulted in six conditions, as follows: two tasks (EF and BF) × three rotation amplitudes (50, 60 and 70 deg). Five repeats of each condition were performed, resulting in 30 trials.

### Analysis

All data were analysed using Matlab (Mathworks Inc.). Arm displacement was calculated as the difference in arm‐on‐body position between the beginning and end of chair movement (mean yaw angle during 500 ms window pre‐ and postmovement). Arm velocity was derived by differentiating position before filtering (low‐pass, 5 Hz, fourth order, zero‐phase‐shift, Butterworth). Peak velocity was measured for each trial.

For statistical analysis of arm displacement and peak velocity, repeated‐measures ANOVA was used to test for main effects and interactions. Significance was set at *P* < 0.05. All statistical tests were performed using SPSS Statistics Version 19 (IBM, Armonk, NY, USA).

## Results

### Experiment 1

**Figure 2:**
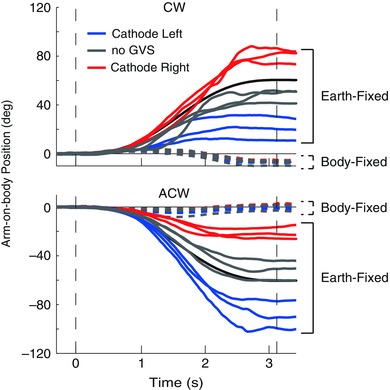
Experiment 1: representative arm kinematics Traces depict arm‐on‐body orientation during 60 deg chair rotations. Positive values indicate leftward motion of the arm on the body. The chair orientation (in space) is shown by the continuous black traces, which have been flipped vertically to aid comparison. Hence, a perfect compensatory movement during the earth‐fixed condition corresponds here to an arm movement trace being identical to chair orientation. In contrast, a trace remaining at zero indicates that the arm remains completely fixed to the body during rotation. Vertical dashed lines indicate rotation onset and end. Note that in the body‐fixed task, GVS conditions are overlapping.

Arm‐on‐body kinematics from a representative participant are shown in Fig. 2 for the 60 deg rotation. The no‐GVS condition is shown by the grey continuous and dashed lines, representing the EF and BF tasks, respectively. During the BF task, the arm hovers around zero, indicating that it remained fixed with respect to the body. In contrast, during the EF condition the arm moves with respect to the body to compensate for chair rotation. This indicates that the subject moved within the appropriate reference frames. However, in the absence of GVS they exhibited a tendency to undercompensate by 10–20 deg in the EF task. When GVS was applied, it had markedly different effects during the two tasks. For the EF task, it increased the amplitude of arm movement when chair movement was directed towards the cathodal ear (Fig. 2, red continuous lines for CW and blue continuous lines for ACW). When chair movement was directed away from the cathode, the compensatory arm movement was smaller. In contrast, GVS had no effect during the BF task.

**Figure 3:**
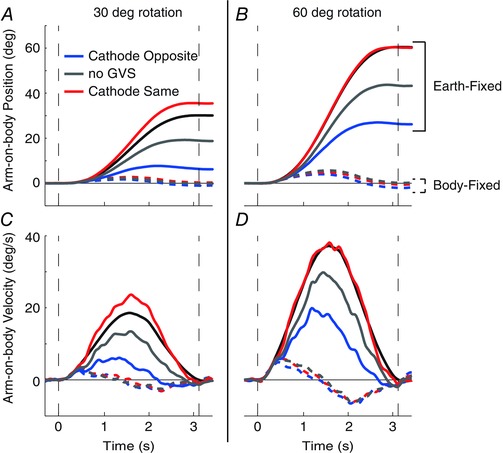
Experiment 1: mean arm kinematics Traces depict mean arm‐on‐body position and velocity for both rotation amplitudes. Anticlockwise (ACW) data have been reversed before combining with clockwise (CW) data. Positive values indicate leftward arm movement during CW rotations (and rightward arm movement during ACW rotations). Chair position and velocity are also shown in continuous black for comparison. Vertical dashed lines indicate rotation onset and end. Note that in the body‐fixed task, GVS conditions are overlapping.

**Figure 4:**
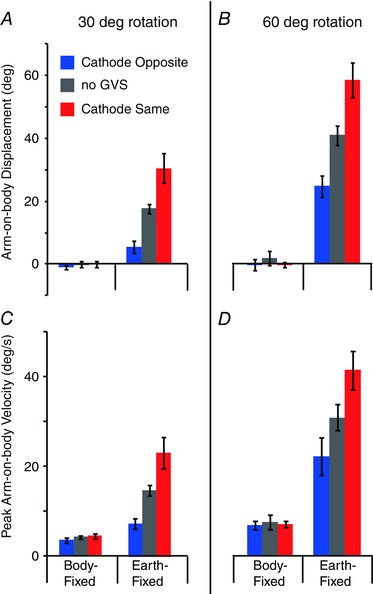
Experiment 1: mean arm displacement and peak velocity Arm displacement was calculated as the difference in arm‐on‐body orientation between the beginning and the end of the trial. Peak velocity was taken as the maximal value of the differentiated position trace during the movement.

The effects observed in the representative subject can be seen on the mean traces in Fig. 3, where arm velocity is also shown. Given that rotation direction produced no significant effects upon arm displacement or peak velocity (*F*_1,7_ ≤ 0.7; *P* ≥ 0.47), both directions were combined after flipping ACW traces. Galvanic vestibular stimulation polarity is now referred to in terms of the cathodal electrode, being either on the same side or opposite to chair rotation direction. The slight velocity deflections apparent across all BF trials are consistent with inertial effects. During EF trials, the tendency to undercompensate without GVS, seen in the individual subject, is clearly apparent; mean undershoot is 12.4 ±^ ^4.0 and 19.4 ± 8.5 deg for 30 and 60 deg rotations, respectively. This tendency causes GVS to improve performance during conditions where the cathode is on the same side as chair rotation (red traces in Fig. 3). Mean arm displacement and peak velocity are both significantly affected by task (Fig. 4; EF *vs*. BF; *F*_1,7_ ≥ 76.7; *P* < 0.001). Crucially, they both exhibit a significant interaction between task and GVS polarity (*F*_2,14_ ≥ 11.1; *P* < 0.001). Individual ANOVAs reveal that this interaction is attributable to the presence of a GVS effect during the EF task (*F*_2,14_ ≥ 13.3; *P* < 0.01) but not the BF task (*F*_2,14_ ≤ 1.9; *P* > 0.18).

Galvanic vestibular stimulation evoked greater arm deviation for the 60 deg rotation [deviation from no‐GVS baseline during EF trials, polarities combined: 16.9 ±^ ^2.8 (60 deg) *vs*. 12.5 ±^ ^2.7 deg (30 deg); *t* = 6.4; *P* < 0.001]. However, in terms of percentage change, the effect of GVS was considerably smaller for the 60 deg rotation [41.6 ± 5.8 (60 deg) *vs*. 72.2 ± 14.0% (60 deg); *t* = 3.4; *P* = 0.012]. Peak arm velocity showed a similar pattern, with rotation amplitude having no influence upon absolute difference [9.6 ± 2.5 (60 deg) *vs*. 8.2 ± 1.3 deg s^−1^ (30 deg); *t* = 0.72; *P* = 0.5] but having a large effect upon percentage difference [33.5 ± 7.7 (60 deg) *vs*. 61.3 ± 11.3% (60 deg); t = 2.9; *P* = 0.022]. This suggests that GVS summates with, rather than multiplies, real movement sensations.

### Experiment 2

**Figure 5:**
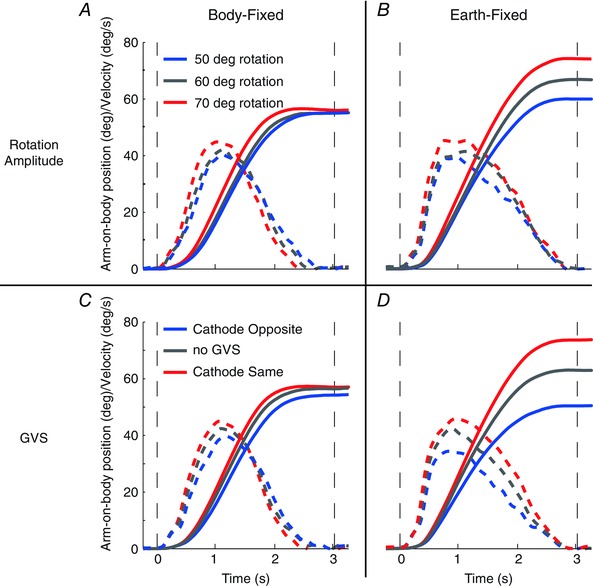
Experiment 2: effects of altered rotation amplitude and GVS upon arm control Traces depict mean arm‐on‐body position (continuous lines) and velocity (dashed lines) for rotation amplitude conditions (*A*, body fixed; and *B*, earth‐fixed) and GVS conditions (*C*, body fixed; and *D*, earth fixed). Vertical dashed lines indicate rotation onset and end.

**Figure 6:**
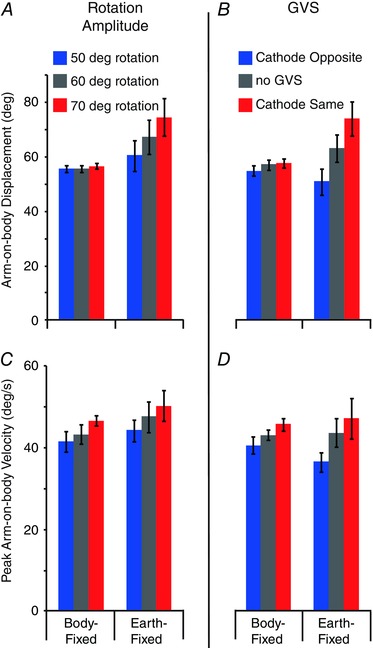
Experiment 2: mean arm‐on‐body displacement and velocity Arm displacement was calculated as the difference in arm‐on‐body orientation between the beginning and the end of the trial. Peak velocity was taken as the maximal value of the differentiated position trace during the movement.

Experiment 2 addressed the confound of different arm kinematics between BF and EF tasks. Here, the tasks were altered to produce a similar arm‐on‐body movement. To determine whether subjects were performing in the correct reference frame, we first randomly altered chair rotation amplitude between 50, 60 and 70 deg in the absence of GVS. This should affect arm‐on‐body motion only during the EF but not the BF task. However, two (of eight) subjects did exhibit a significant difference in hand displacement in the BF task (*F*_2,8_ ≥ 4.91; *P* ≤ 0.041) and were therefore excluded from further analysis. Mean kinematics for the remaining subjects are shown in Fig. 5 *A* and *B*. For arm displacement, there is an interaction between rotation amplitude and task (Fig. 6 *A*; *F*_2,10_ = 34.1; *P* < 0.001). This is attributable to compensatory arm movements being larger with increasing chair motion during the EF (*F*_2,10_ = 34.7; *P* < 0.001) but not the BF task (*F*_2,10_ = 1.0; *P* = 0.40). However, peak arm velocity does not exhibit the same interaction (Fig. 6 *C*; *F*_2,10_ = 0.23; *P* = 0.80) and is significantly affected by rotation amplitude during both tasks (*F*_2,10_ ≥ 4.13; *P* ≤ 0.049). The reason for this displacement–velocity discrepancy can be seen in the mean velocity traces for the BF task (dashed traces in Fig. 5 *A*). During the 70 deg rotation, arm velocity is initially higher (than the 60 deg rotation) but then immediately reduces for the remainder of the movement. The reverse is true for the 50 deg rotation. Thus, while peak arm velocity is affected by rotation amplitude during the BF task, final arm position is preserved. This indicates that subjects moved in the appropriate reference frame.

For these well‐behaved subjects, the effects of GVS were studied in a separate block of trials, with arm movement shown in Fig. 5 *C* and *D*. During the baseline no‐GVS condition, peak velocities are closely matched between BF and EF tasks [43.0 ± 3.7 (BF) and 43.6 ± 9.8 deg s^−1^ (EF); see also grey bars in Fig. 6 *D*]. Despite this, GVS had different effects upon arm displacement (GVS–task interaction: *F*_2,10_ = 18.21; *P* < 0.001). A breakdown of this interaction confirms that GVS had no effect upon arm displacement during the BF task (Fig. 6 *B*; *F*_2,10_ = 3.89; *P* = 0.057). This contrasts with the EF task, where compensatory arm movements were larger (smaller) with the cathode on the same (opposite) side as chair motion (*F*_2,10_ = 15.08; *P* < 0.001). However, similar to the effect of rotation amplitude, peak arm velocity was significantly affected by GVS during both tasks (Fig. 6 *D*; *F*_2,10_ ≥ 6.12; *P* ≤ 0.018). In this case, peak arm velocity was higher during cathode‐same trials and smaller during cathode‐opposite trials. Again, during the BF task these changes were immediately compensated by velocity reversals, thus preserving final arm position (dashed traces in Fig. 5 *C*).

## Discussion

Galvanic vestibular stimulation, when applied concurrently with real motion, caused deviations in arm orientation when attempting to point towards an earth‐fixed target. These deviations mimicked those evoked by real chair rotation; hence, they reflect a mechanism that uses vestibular feedback to compensate for unpredictable body motion. The absence of compensatory arm movements when reaching in a body‐centred reference frame emphasizes the purpose of the mechanism. In this case, body motion is no longer relevant for movement accuracy and no compensation is required.

During earth‐fixed pointing, subjects consistently undercompensated in the absence of GVS. In the first experiment, arm movement was around two‐thirds the magnitude of chair rotation. This cannot be attributed to biomechanical limitations, because the arm was capable of moving further when GVS was applied. Although the precise cause is unclear, underestimation of whole‐body motion perception for velocities greater than ∼10 deg s^−1^ has been previously reported (Day & Fitzpatrick, 2005). Peak chair velocity here was always in excess of this value, being ≥18 deg s^−1^. The consequence of the undercompensation was that GVS genuinely improved performance above baseline during the EF task.

In experiment 1, when subjects attempted to point continuously at an earth‐fixed location whilst being rotated, compensatory arm movements were systematically modified by GVS. With the cathode on the same side as chair rotation, arm movements were larger. With reversed electrode polarity or rotation direction, GVS made arm movements smaller. These observations can be interpreted in the context of established perceptual effects of GVS. Day & Fitzpatrick (2005) studied rotation perception using similar methodology, applying GVS concurrently with real rotation. When they asked subjects to return themselves to the perceived starting point, the pattern of errors revealed that GVS increased or decreased movement sensation, depending on polarity. For example, with the head tilted down and the cathode over the right ear, subjects perceived a clockwise rotation as being further than really experienced. In the same conditions of head posture, polarity and rotation direction, our results show that GVS increased the magnitude of the compensatory arm movement. Specifically, it caused the arm to move further in a direction opposite to chair rotation. The magnitude of the evoked arm movement was similar for 30 and 60 deg rotations, suggesting that the sensory effect of GVS summates with, rather than multiplies, real movement sensations. To ensure that the stimulating current affected rotation sensation, we tilted the head down such that Reid's plane was ≥45 deg below horizontal. This was done to align the GVS head rotation vector closer towards the axis of chair rotation (Reynolds & Osler, 2012). Given that this vector was ∼20 deg below Reid's plane (in head co‐ordinates; Day & Fitzpatrick, 2005; Osler & Reynolds, 2012), this places the GVS vector ∼25 deg above vertical, or less (in room co‐ordinates). By taking the cosine of this angle, we estimate that this head orientation would produce ∼91% of the maximal possible effect of GVS upon yaw rotation perception. Thus, although we did not measure movement sensation or perception, our experiment was deliberately engineered to produce the largest influence of GVS upon yaw rotation sensation. The results are therefore consistent with an arm movement that compensates for this false sense of body movement.

In the first experiment, when subjects were asked to point within a body‐fixed reference frame, GVS had no effect. In this case, they simply maintained their outstretched arm fixed with respect to their body. As they were rotated, GVS was applied but did not influence final arm position. Abolition of the response in this way supports the idea of a functional mechanism linking vestibular feedback to arm control. For example, when scratching our nose while standing, we must control the arm in a body‐fixed reference frame. There is no need to compensate for body sway; this might direct a finger inappropriately into an eye. In contrast, when reaching for a handrail while standing, the arm must take into account (and compensate for) any ongoing body movement to reach its intended target. Our results show that a simple change in task goal is sufficient to reduce the effect of vestibular input on arm movement.

However, the difference between the EF and BF tasks of experiment 1 might not be attributable to different reference frames *per se*, but to the profoundly different movement kinematics required of the two tasks. The BF task involved zero arm‐on‐body movement and might be considered a non‐task. This lack of movement could be responsible for the absence of a GVS effect, in itself. Experiment 2 was designed to address this confound by attempting to match arm‐on‐body kinematics. The results corroborated those of the first experiment. Final arm orientation was altered by GVS during the EF but not the BF task, despite arm‐on‐body velocity being matched between tasks. Furthermore, experiment 2 confirmed that the effect of GVS was very similar to that caused by real changes in rotation amplitude. Figure 5 *A–D* shows that arm kinematics are similar in both quality and quantity for both interventions. This similarity confirms that arm movements evoked by GVS reflect a mechanism compensating for a sensation of altered rotation amplitude. But while GVS did not affect final arm displacement during the BF task, the same is not true for peak arm velocity. The dashed red trace in Fig. 5 *C* shows that, with the cathode electrode on the same side as chair movement, peak arm velocity increased slightly. This was immediately followed by a decrease in velocity, such that final arm position was unaffected. Swapping polarity caused the opposite effect (dashed blue trace in Fig. 5 *C*). This suggests that, although the overall movement goal was successfully achieved in body‐fixed co‐ordinates, there was a tendency to act in earth‐fixed co‐ordinates during the early phase of the movement, despite the instruction.

This was also apparent during real changes in chair rotation (dashed traces in Fig. 5 *A*). During real changes in chair rotation, inertial effects could explain this tendency; as the chair rotates faster, the inertia of the arm might result in greater arm‐on‐body movement. However, this cannot explain the effect of GVS upon arm velocity. In this case, the mechanics are identical, the only change being sensory input. This therefore suggests a general tendency to act in an earth‐fixed reference frame during the ballistic phase of an arm movement, even when the final goal of that movement is defined in body co‐ordinates. This early component of the arm response may be immune to cognitive influence, as has been suggested for body sway responses (Guerraz & Day, 2005; Reynolds, 2010). Nevertheless, this tendency was very small in comparison to the overall kinematic differences between BF and EF tasks; final arm position was ultimately maintained during the BF task. Experiment 2 therefore confirms that vestibular control of the upper limb can be modified purely by changing the task goal.

Previous research has shown GVS to influence upper‐limb movement in a variety of body postures. This occurs when reaching towards earth‐fixed targets, during both sitting (Bresciani *et al*. 2002 *b*; Mars *et al*. 2003; Moreau‐Debord *et al*. 2014) and standing (Bresciani *et al*. 2002 *a*). It has also been demonstrated when maintaining arm position in space, similar to the present experiments, but while seated in a fixed chair (Pu *et al*. 2012). As shown here, the direction of the evoked arm movement is consistently towards the anode electrode, with the largest magnitude observed during head‐down tilt, being minimal when the head is upright (Moreau‐Debord *et al*. 2014). This dependence on head orientation is consistent with GVS producing a sensation of head roll about a naso‐occipital axis in the direction of the cathode electrode (Fitzpatrick *et al*. 2006; Reynolds, 2011; Reynolds & Osler, 2012). Our interpretation is that this arm movement is a counteractive response to sensed body motion, to maintain arm trajectory in space. But if so, the response should scale with perception of body motion. Specifically, it should be attenuated when body motion perception is suppressed. Such attenuation has been demonstrated for postural responses to GVS, where response magnitude is inversely related to the availability of veridical sensory information (Day *et al*. 2002). For example, opening the eyes or making light contact with an earth‐fixed object both attenuate the sway response to GVS (Britton *et al*. 1993; Day & Guerraz, 2007). Likewise, sway responses are suppressed when seated *vs*. standing (Fitzpatrick *et al*. 1994). This suggests that postural responses are highly task dependent, occurring only when two conditions are met: firstly, the stimulus must evoke a sense of body motion; and, secondly, this motion must have a consequence for task performance (balancing or reaching). However, when we consider the upper limb, the existing literature is ambiguous on this crucial point. In particular, Bresciani *et al*. (2002 *a*,*b*) found that GVS‐evoked perturbations of reaching were of similar magnitude when standing *vs*. sitting. Unlike here, during their seated condition the chair was fixed in space, precluding any possibility of body movement. Other authors have also demonstrated significant effects of GVS upon arm movement while sitting in fixed chairs (Pu *et al*. 2012; Moreau‐Debord *et al*. 2014). This apparent invariance of the response with the task could be interpreted as the manifestation of a ‘hard‐wired’ reflex; that is, a fixed relationship between vestibular input and upper‐limb output, regardless of task or posture. This seems unlikely because most vestibular reflexes do exhibit task dependence (Forbes *et al*. 2014 *a*), with the exception of the vestibulocollic reflex, which is permanently engaged (Forbes *et al*. 2014 *b*).

One clue to this anomaly may be the magnitude of the reported responses. In our data, GVS altered arm orientation by up to 17 deg compared with the no‐GVS condition (equating to 177 mm for an arm of length 600 mm). Previously reported effects are at least one order of magnitude lower than this (≤1.56 deg in Bresciani *et al*. 2002 *a*,*b*; ≤23 mm in Mars *et al*. 2003; and ≤68 mm in Moreau‐Debord *et al*. 2014). Why the discrepancy? One trivial explanation might be task duration. In our study, it was 3 s, *vs*. ≤1 s in the above‐mentioned literature. This would allow more time for GVS to deviate arm position. However, Pu *et al*. (2012) exposed seated subjects to continuous GVS for at least 10 s and observed only ∼10 mm deviation, suggesting this is not the explanation. The likely cause is differences in posture and task. In all of these studies, the head was fixed in space, precluding the possibility of any head and/or body motion, with the exception of Mars *et al*. (2003), where subjects sat on a stool with the trunk free. In the case of Bresciani *et al*. (2002 *a*,*b*), a bite‐bar was used to constrain the head, during both sitting and standing. This would provide both mechanical stability and sensory input. In this case, the difference in posture between standing and sitting might be irrelevant, because the body motion that normally accompanies a standing posture was prevented. This may explain why there was minimal difference between the two postures and would also explain the relatively small influence of GVS. In our experiment, the body was rotating in space, and GVS was superimposed upon this natural motion. During the earth‐fixed condition, this caused a relatively large change in arm trajectory. This is likely to result from the paucity of veridical sensory information, which would otherwise conflict with GVS‐evoked sensations, i.e. there was no contact with earth‐fixed objects. It therefore seems logical to interpret the observed arm movements as compensatory responses to this false sense of body motion.

## Additional information

### Competing interests

None declared.

### Author contributions

This study was performed at the School of Sport, Exercise and Rehabilitation sciences, University of Birmingham, UK. C.P.S. and R.F.R. contributed to conception and design of the experiments; analysis and interpretation of data; drafting the article; and revising it for important intellectual content. C.P.S. collected and assembled data. Both authors approved the final version of manuscript and agree to be accountable for all aspects of the work in ensuring that questions related to the accuracy or integrity of any part of the work are appropriately investigated and resolved. All persons designated as authors qualify for authorship, and all those who qualify for authorship are listed.

### Funding

This work was supported by a Biotechnology and Biological Sciences Research Council (BBSRC) Industry Interchange Award to R.F.R. C.P.S. is supported by the BBSRC Midlands Integrative Biosciences Training Partnership doctoral programme.
